# Capsule Type of *Streptococcus pneumoniae* Determines Growth Phenotype

**DOI:** 10.1371/journal.ppat.1002574

**Published:** 2012-03-08

**Authors:** Lucy J. Hathaway, Silvio D. Brugger, Brigitte Morand, Mathieu Bangert, Jeannine U. Rotzetter, Christoph Hauser, Werner A. Graber, Suzanna Gore, Aras Kadioglu, Kathrin Mühlemann

**Affiliations:** 1 Institute for Infectious Diseases, University of Bern, Bern, Switzerland; 2 Institute of Infection and Global Health, University of Liverpool, Liverpool, United Kingdom; 3 Liverpool School of Tropical Medicine, Liverpool, United Kingdom; 4 Anatomical Institute, University of Bern, Bern, Switzerland; 5 Department of Infectious Diseases, University Hospital, Bern, Switzerland; The University of Texas Health Science Center at San Antonio, United States of America

## Abstract

The polysaccharide capsule of *Streptococcus pneumoniae* defines over ninety serotypes, which differ in their carriage prevalence and invasiveness for poorly understood reasons. Recently, an inverse correlation between carriage prevalence and oligosaccharide structure of a given capsule has been described. Our previous work suggested a link between serotype and growth *in vitro*. Here we investigate whether capsule production interferes with growth *in vitro* and whether this predicts carriage prevalence *in vivo*. Eighty-one capsule switch mutants were constructed representing nine different serotypes, five of low (4, 7F, 14, 15, 18C) and four of high carriage prevalence (6B, 9V, 19F, 23F). Growth (length of lag phase, maximum optical density) of wildtype strains, nontypeable mutants and capsule switch mutants was studied in nutrient-restricted Lacks medium (MLM) and in rich undefined brain heart infusion broth supplemented with 5% foetal calf serum (BHI+FCS). In MLM growth phenotype depended on, and was transferred with, capsule operon type. Colonization efficiency of mouse nasopharynx also depended on, and was transferred with, capsule operon type. Capsule production interfered with growth, which correlated inversely with serotype-specific carriage prevalence. Serotypes with better growth and higher carriage prevalence produced thicker capsules (by electron microscopy, FITC-dextran exclusion assays and HPLC) than serotypes with delayed growth and low carriage prevalence. However, expression of *cpsA*, the first capsule gene, (by quantitative RT-PCR) correlated inversely with capsule thickness. Energy spent for capsule production (incorporation of H3-glucose) relative to amount of capsule produced was higher for serotypes with low carriage prevalence. Experiments in BHI+FCS showed overall better bacterial growth and more capsule production than growth in MLM and differences between serotypes were no longer apparent. Production of polysaccharide capsule in *S. pneumoniae* interferes with growth in nutrient-limiting conditions probably by competition for energy against the central metabolism. Serotype-specific nasopharyngeal carriage prevalence *in vivo* is predicted by the growth phenotype.

## Author Summary

*Streptococcus pneumoniae* bacteria are responsible for serious human infections including meningitis, pneumonia and bacteraemia and are a common cause of otitis media (ear infection) in children. However, they most often reside harmlessly in the infant nasopharynx. An association has long been observed between the type of polysaccharide capsule surrounding the bacteria and harmless colonization versus invasive disease. Here we suggest that capsule types that are costly for the bacteria to make are produced in lower quantities and their production limits the growth of the bacteria in nutrient-restricted conditions. In contrast, bacteria with capsules that require less energy can produce more capsule and grow more successfully. This may be an explanation for why *S. pneumoniae* with certain capsule types can be effective long-term colonizers of the nasopharynx while others need a richer nutritional environment to flourish and so are most often associated with invasive disease. This information may be of use when considering which capsules types to target in future vaccines.

## Introduction

*Streptococcus pneumoniae* is a major pathogen causing several serious human diseases including pneumonia, meningitis and sepsis as well as being a common cause of otitis media. The bacteria are usually surrounded by one of at least 92 known varieties of polysaccharide capsule [1], [2] which acts as the most important virulence factor by protecting the bacteria from destruction by host phagocytes [3], [4]. On the basis of the reaction between the polysaccharide capsule and specific antibody, *S. pneumoniae* is classified by serotype. Whilst some serotypes have a tendency to colonize the nasopharynx more often asymptomatically, others are found to be carried less frequently but are associated with invasive and mucosal disease [5]–[7]. The reasons for this difference between serotypes have not been clearly established.

Our previous work suggested a link between colonization prevalence *in vivo*, capsule type and growth *in vitro* [8]–[10]. Weinberger et al. [7] have suggested that increased carriage prevalence is associated with heavier encapsulation for protection from neutrophil-mediated killing. This group also found an inverse correlation between carriage prevalence and the predicted metabolic burden of capsule type, based on the number of carbons and the number of high-energy bonds required to generate one polysaccharide repeat unit.

Little is known about the nutritional environment which is encountered by *S. pneumoniae* in the nasopharynx but we propose that when comparing phenotypes of different serotypes *in vitro* it may be important to consider their behaviour in a relatively nutritionally poor environment. This might more closely reflect the nasopharynx than very rich media commonly used for *in vitro* culture. For example, albumin concentration in the fluid layer covering the epithelium in the lower respiratory tract is approximately 10-fold lower than serum albumin concentration [11]. Here, we investigated whether the burden of capsule production manifested itself as a delay or reduction in growth of the bacteria under conditions of restricted nutrition as might be experienced in the nasopharynx. The use of 81 different capsule switch mutants allowed us to exclude any effects of the genetic background. Our results suggest that, indeed, capsules of low prevalence serotypes do delay and reduce growth and that this effect is transferrable with the capsule operon. We found a significant correlation between carriage prevalence of serotypes and their growth phenotype. We also found a relationship between amount of capsule produced and serotype: High prevalence serotypes making more capsule than low prevalence serotypes in nutrient-restricted conditions.

## Results

### Capsule types exhibit distinct growth patterns which are transferred with capsule genes

**Figure 1:**
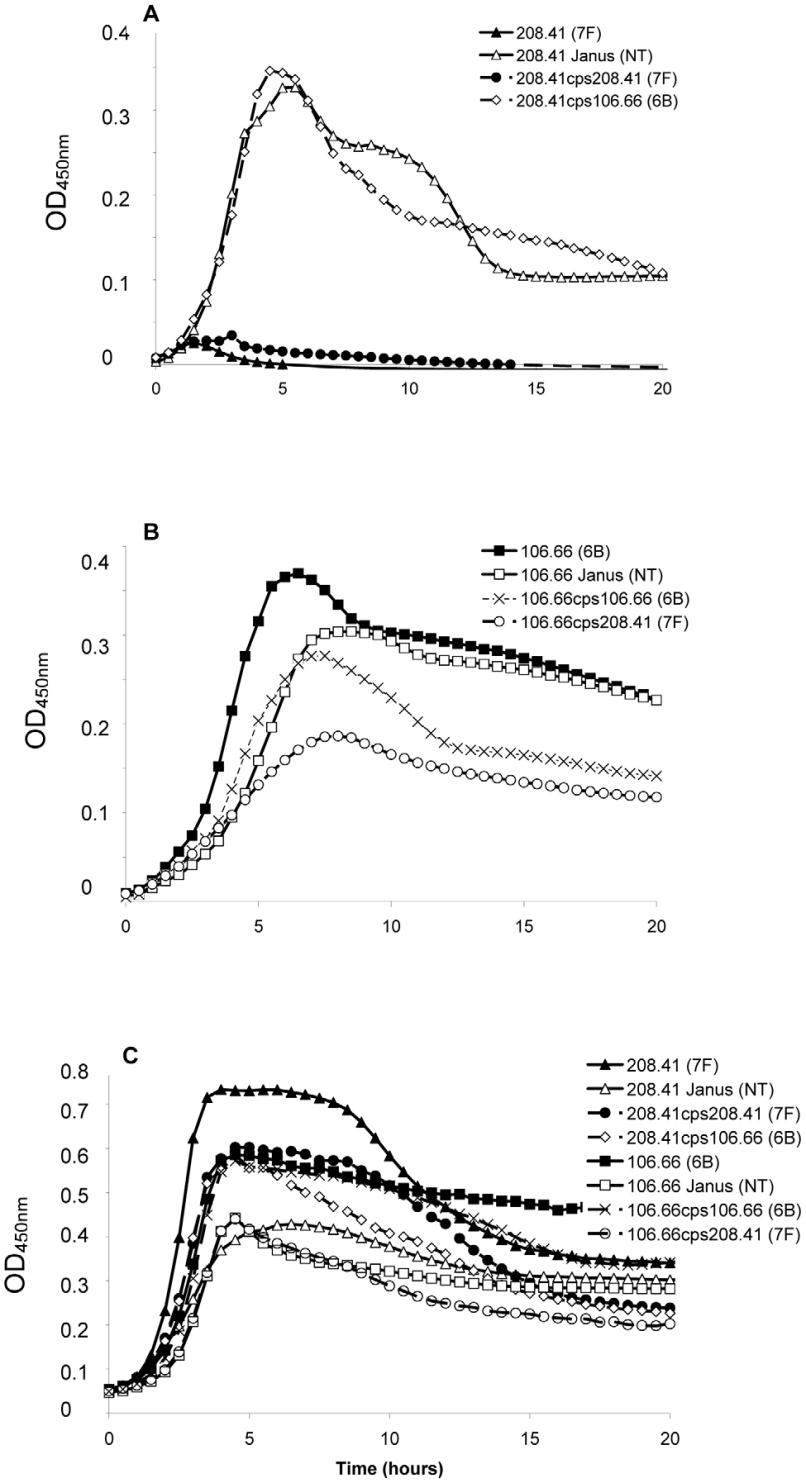
Growth patterns of strain 208.41 of serotype 7F and strain 106.66 of serotype 6B and their capsule switch mutants. A) Loss of 7F capsule greatly increases growth and acquisition of a 6B capsule has no effect on lag phase compared to the nontypeable Janus mutant in MLM. Reacquisition of the 7F capsule reduces growth to that of the wildtype 7F strain. B) Loss of 6B capsule reduces maximum OD_450nm_ and slightly increases lag phase in MLM. Acquisition of a 7F capsule further enhances these effects. Reacquisition of the 6B capsule led to a lag phase between that of the wildtype 6B strain and the Janus mutant. C) In BHI+FCS medium there is little difference in lag phase between the strains or maximum OD_450 nm_ between the wildtype strains. Graphs are representative of three independent experiments.

Strains representing the low carriage serotypes 4, 7F, 14, 15, and 18C and the high carriage prevalence capsule types 6B, 9V, 19F, and 23F were used. Growth over 20 hours was monitored for wildtype strains, non-encapsulated mutants in which the capsule operon was replaced by the Janus cassette and capsule switch mutants using a nutrient-limited modified Lacks medium (MLM) and the rich non-defined BHI+FCS medium. Experiments using capsule switch pairs of two 7F and one 6B strain demonstrated that serotype 7F and 6B exhibit different growth patterns, which could be transferred with the capsule operon (Figure 1 and Figure S1). In MLM, the 7F wildtype barely grew but loss of the 7F capsule operon allowed enhanced growth and this was only slightly reduced by acquisition of the 6B capsule (Figure 1A). In contrast, the wildtype 6B strain grew well in the MLM but loss of capsule or replacement with the 7F capsule reduced and delayed growth (Figure 1B). However, such growth differences were not apparent in the rich BHI+FCS medium (Figure 1C).

**Figure 2:**
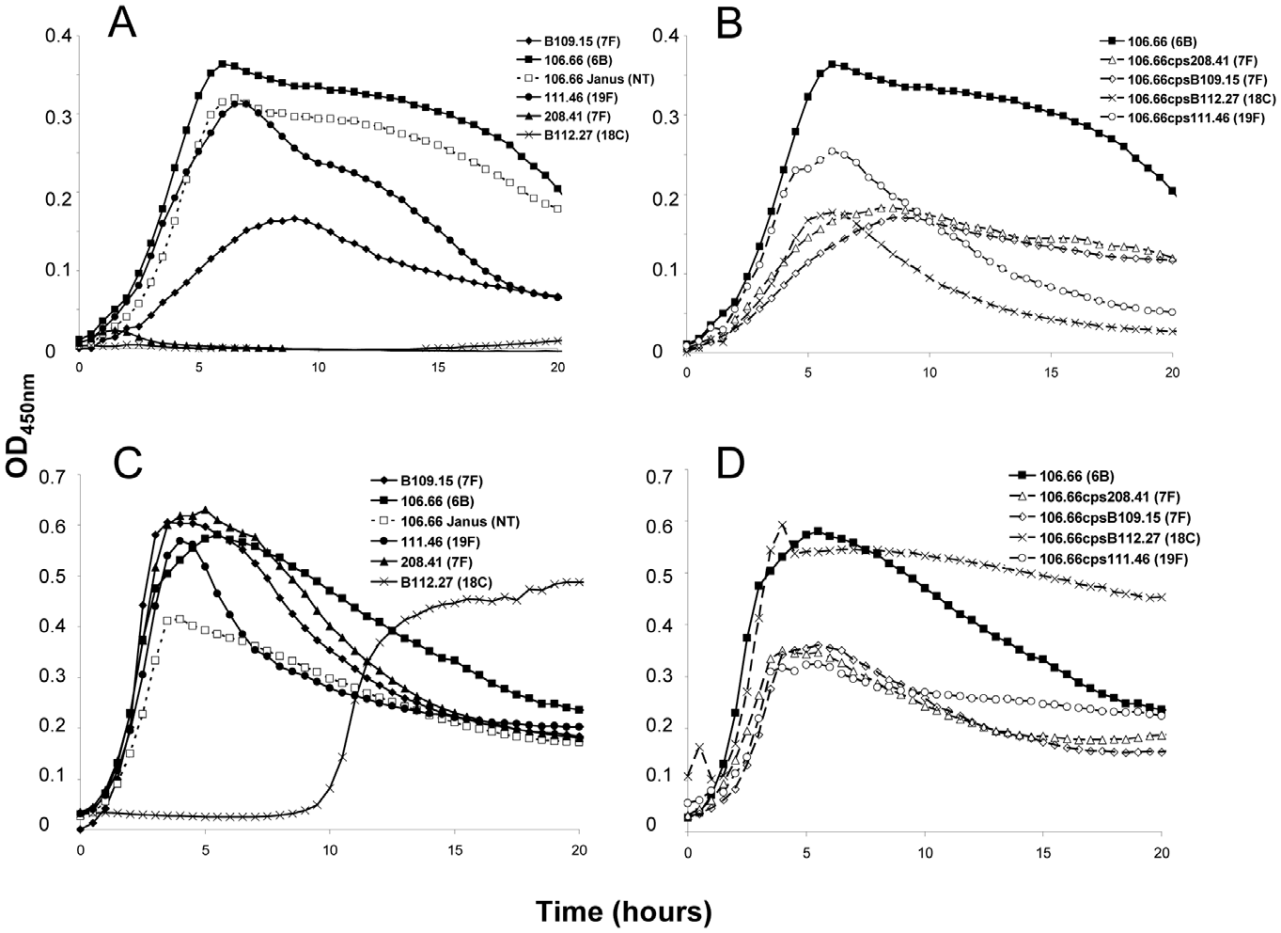
Growth curves of strain 106.66 (serotype 6B) after replacing 6B capsule operon with operons of different serotypes. Growth of the wildtypes and nontypeable Janus mutant (A and C) and strain 106.66 with its capsule replaced by that of serotype 7F, 18C or 19F strains (B and D) was measured in MLM (A and B) and BHI+FCS medium (C and D). In MLM, growth of 106.66 was reduced and delayed by acquisition of other capsules. The effect was less for 19F than 7F or 18C reflecting the growth pattern of the wildtype donors. Graphs are representative of three independent experiments.

In order to extend this observation to more serotypes, but exclude an effect of the chromosomal background, growth patterns were studied for capsule switch mutants of two 7F strains, an 18C strain and a 19F strain in the same 6B background. Growth was measured quantitatively by measuring the length of the lag phase (until reaching an OD_450 nm_ of 0.3) and the maximum OD_450 nm_. The results showed that serotype 6B wildtype strain had a shorter lag phase and grew to a higher OD_450 nm_ than wildtype strains of serotypes 7F, 18C and 19F in MLM (Figure 2A). Following replacement of the 6B capsule with 7F, 18C or 19F capsules, growth of capsule switch mutants in MLM was reduced relative to the 6B wildtype. Growth was enhanced for capsule switch mutants of serotypes 7F and 18C strains relative to their donor strains and growth was similar for the 19F capsule switch mutant and the donor strain (Figure 2B). This effect was not seen in BHI+FCS medium where growth of all wildtype strains was similar (with the exception of the serotype 18C strain which had a prolonged lag phase in this medium) (Figure 2C). In BHI+FCS, the capsule switch mutants grew to a lower OD than the wildtype 6B strain with the exception of the recipient of the 18C capsule which grew as well as the 6B wildtype (Figure 2D).

**Figure 3:**
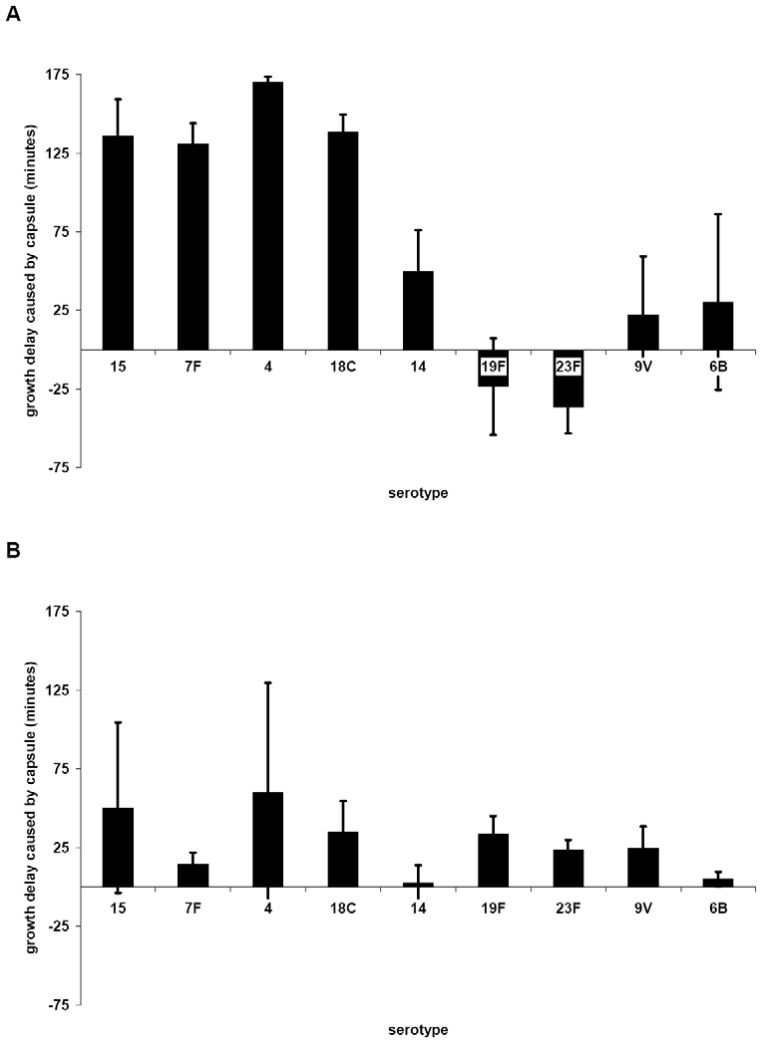
Influence of capsule serotype on length of lag phase of growth in MLM (A) and BHI+FCS (B). Change in growth due to the capsule type was calculated as the difference in time to reach an OD_450 nm_ of 0.3 between the non-encapsulated Janus mutant and the strain of the same genetic background but with a new capsule. The average values for each serotype are plotted; error bars represent one standard error. (For serogroup 15 n = 3, for 7F n = 10, for 4 n = 2, for 18C n = 12, for 14 n = 9, for 19F n = 11, for 23F n =  12, for 9V n = 9, for 6B n = 5). The greatest delay of growth in MLM was caused by capsules of serotypes 15, 7F, 4 and 18C, and to a lesser extent 14.

In order to determine the effect of additional serotypes, the growth of 10 strains in which the capsule operon had been replaced by the Janus cassette was compared with that of their 10 parent strains (Table S1) and with that of 81 capsule switch mutants in which a new capsule operon (serotypes 4, 6B, 7F, 9V, 14, 15, 18C, 19F, 23F) was inserted in place of the Janus cassette (Table S2). Change in growth due to the capsule type was calculated as the difference in time to reach an OD_450 nm_ of 0.3 between the non-encapsulated Janus mutant and the strain of the same genetic background but with a new capsule. The average values of these changes in growth in MLM due to capsules of different serotypes are shown in Figure 3A. The greatest growth delays in MLM were due to capsules of serotypes 15, 7F, 4 and 18C and, to a lesser extent, serotype 14. Capsules of serotypes 19F, 23F, 9V and 6B seemed to cause less or no delay in growth. The delay in growth due to capsule was less in BHI+FCS medium and differed less between the serotypes (Figure 3B).

### Capsule type related growth patterns correlate with carriage prevalence

**Figure 4:**
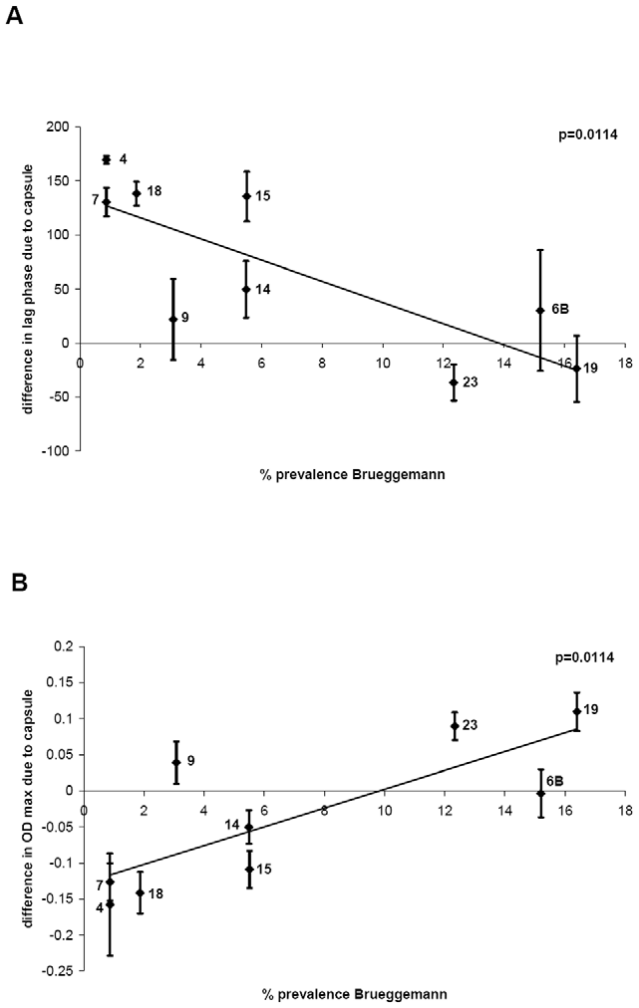
Relationship between growth pattern and carriage prevalence of serotype. A) For each serotype, the delay of growth due to the capsule was plotted against percentage carriage prevalence data obtained from a previous study [5] and an inverse correlation was found, p = 0.0114. B) For each serotype, the difference in maximum OD_450 nm_ due to the capsule was plotted against percentage carriage prevalence data [5] and a positive correlation was found, p = 0.0114.

The average values of changes in growth due to different capsule types (see above and Figure 3) were plotted against the percentage carriage prevalence of serotype determined by the epidemiological study of Brueggemann et al. [5]. Figure 4A shows that there is an inverse correlation between delay of growth due to capsule type and percentage carriage prevalence (p = 0.0114), with serogroup 9 as somewhat of an outlier. The delay in growth due to capsule was less in BHI+FCS medium and differed less between the serotypes (data not shown). A significant inverse correlation was also found when the growth delay was plotted against the percentage carriage prevalence data of a Swiss population [6] (p = 0.0387) and data from an international study [7] (p = 0.0379) (Figure S2).

In a similar manner, the difference in maximum OD_450 nm_ due to the capsule type in MLM was plotted against percentage carriage prevalence data obtained from the study of Brueggemann et al. [5] and a significant a positive correlation was found (p = 0.0114) (Figure 4B). Using the prevalence values of the Swiss study [6] a significant correlation was also observed (p = 0.0361) and using the prevalence values of Weinberger et al. [7] the same trend was observed but narrowly missed being statistically significant (p = 0.0511) (Figure S3).

### Carriage efficiency depends on, and is transferred with, capsule operon

**Figure 5:**
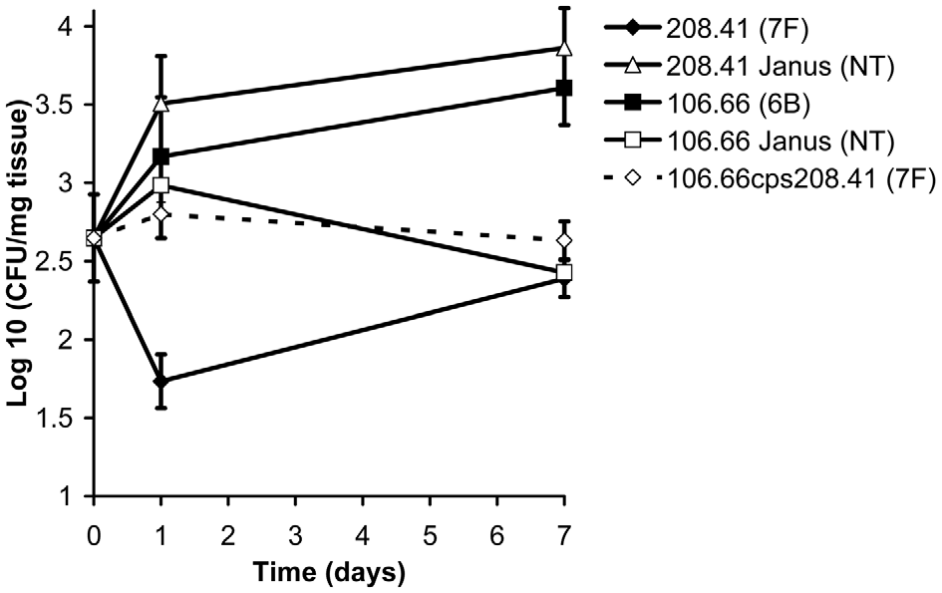
Relationship between capsule type and carriage efficiency in a mouse model. Ability of wildtype strains and mutants to colonize the nasopharynx of female MF1 mice was determined by quantification of colony forming units (CFU) per mg nasopharyngeal tissue on the day of inoculation and 1 and 7 days later. By day 7, the disadvantage of a 7F capsule, but not a 6B capsule, in colonization was apparent. Error bars represent one standard error.

Figure 5 shows the colonization efficiency of different wildtype strains and mutants in the nasopharynx of mice over the course of a week. By day 7 after inoculation, the wildtype serotype 6B strain had established a higher level of colonization than the wildtype 7F strain (p = 0.0017). However, in the mutant in which the 7F capsule had been removed (208.41 Janus), colonization was significantly greater than that of its parental serotype 7F strain (p = 0.0008). In contrast, when the 6B capsule was replaced with a 7F capsule (mutant 106.66cps208.41) colonization decreased significantly compared with that of the 6B parental strain (p = 0.0063). For the serotype 6B strain, loss of the capsule (resulting in 106.66 Janus mutant) was a disadvantage for colonization (p = 0.0011).

### Capsule types differ by their metabolic burden

**Figure 6:**
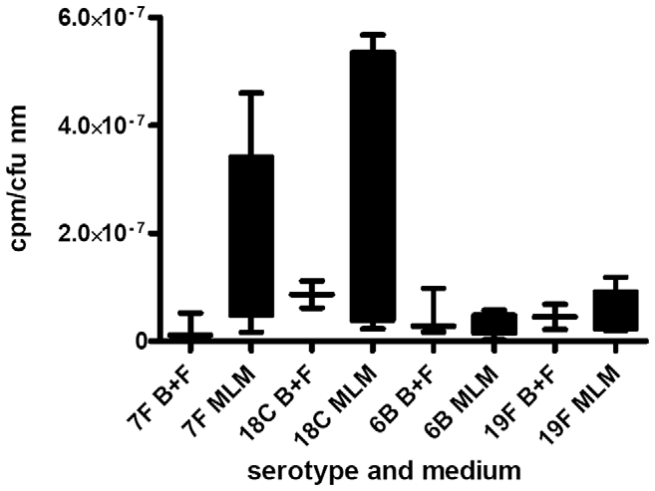
Metabolic burden of capsule was determined by incorporation of 3H-labelled glucose into the capsule. The fraction of labelled glucose taken up which was detected in the capsule was determined for wildtype strains and capsule switch mutants and the data pooled by serotype and normalized for colony forming units and thickness of capsule. Strains with 7F and 18C capsules incorporated a higher proportion of glucose into their capsules than the high colonization prevalence serotypes 6B and 19F in the MLM (p = 0.0068) but in the BHI+FCS medium there was no significant difference between the serotypes (p = 0.9493).

As a measure of the relative metabolic burden of the capsule, the fraction of 3H-labelled glucose taken up and detected in the capsule was determined. Figure 6 shows the results for wildtype strains and capsule switch mutants pooled by serotype and normalized for colony forming units and thickness of capsule. Strains with 7F and 18C capsules incorporated a higher proportion of glucose into their capsules than the high colonization prevalence serotypes 6B and 19F in the MLM (p = 0.0068) but in the BHI+FCS medium there was no significant difference between the serotypes (p = 0.9493).

### There is a relationship between carriage prevalence and capsule thickness in nutrient-restricted medium

**Figure 7:**
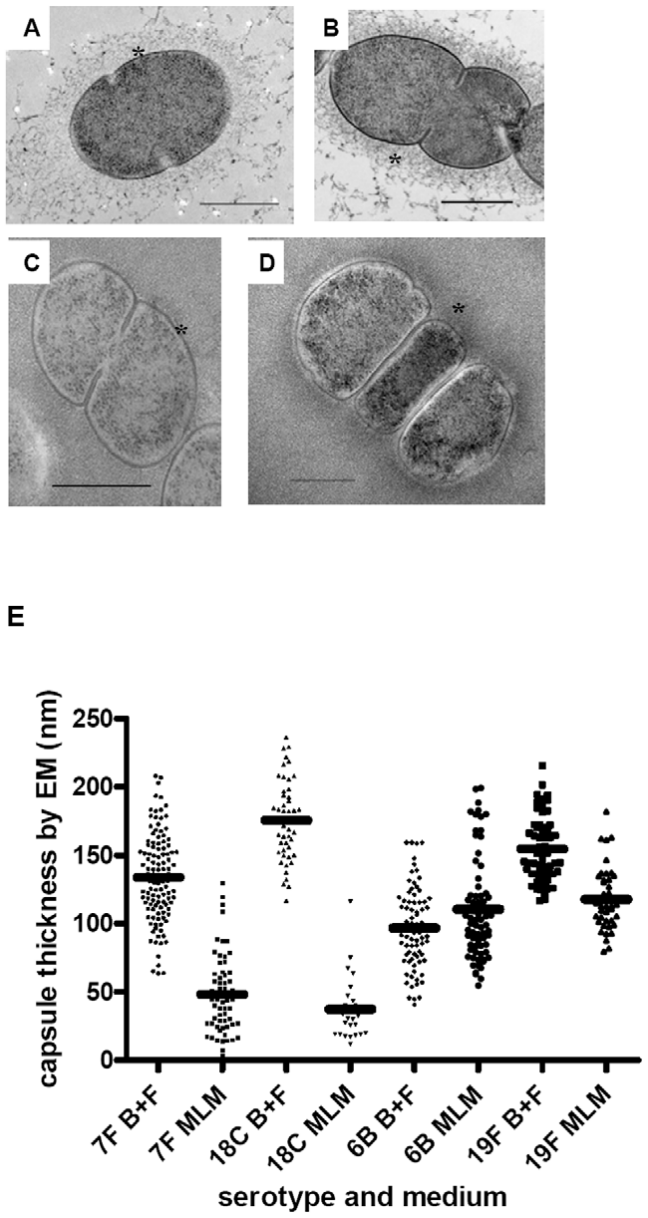
Electron microscopy showing polysaccharide capsules for measuring capsule thickness. In BHI+FCS medium (A and B) strains 208.41 (serotype 7F) (A) and 106.66 (serotype 6B) (B) expressed similar amounts of capsule. In MLM (C and D) thicker capsule can be distinguished for 106.66 (6B) (D) than 208.41 (7F) (C). * indicates capsule. Bar = 500 nm. E) Capsule thickness (nm) in wildtypes and capsule switch mutants in BHI+FCS (B+F) medium and in MLM. Capsules were thicker in BHI+FCS than in MEM (p<0.0001) for all serotypes except 6B. In MLM, the high prevalence serotypes 6B and 19F had thicker capsules than the low prevalence serotypes 7F and 18C (p<0.0001). (n per serotype and medium category = 25–118).

**Figure 8:**
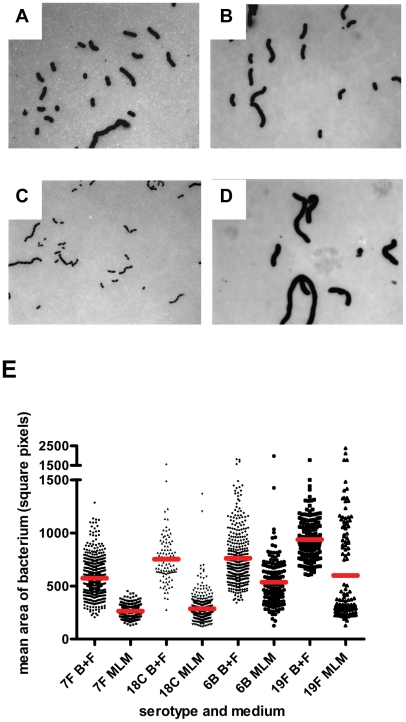
FITC-dextran exclusion assay. In BHI+FCS medium (A and B) strains 208.41 (serotype 7F) (A) and 106.66 (serotype 6B) (B) expressed similar amounts of capsule. In MLM (C and D) strain 106.66 (6B) (D) bacteria are bigger than strain 208.41 (7F) (C). All pictures are to the same scale, original magnification = 630 X. E) Mean area per bacterium (square pixels) was greater in BHI+FCS than in MEM (p<0.0001). In both media bacteria of 6B and 19F serotypes had thicker capsules than bacteria of 7F and 18C serotypes (p<0.0001). (n per serotype and medium category = 96–647).

Next, we investigated whether the relationship between capsule type and growth pattern may be determined by the amount of capsule produced since this poses a metabolic burden to the bacterial cell. Capsule thickness was determined by electron microscopy (EM), FITC-dextran exclusion assay and quantitative HPLC analysis of cell bound capsular polysaccharides in wildtype strains of serotypes 7F (two strains), 18C, 6B and 19F and their six capsule switch mutants (6B background with the two different 7F capsules, 18C or 19F capsule and both 7F backgrounds with the 6B capsule). Examples of the EM images are shown in Figure 7 and for FITC-dextran assay in Figure 8 for a 7F serotype and a 6B serotype.

By EM, it could be seen that in BHI+FCS medium strains 208.41 (serotype 7F) (Figure 7A) and 106.66 (serotype 6B) (Figure 7B) expressed similar amounts of capsule. In MLM thicker capsule can be distinguished for 106.66 (6B) (Figure 7D) than 208.41 (7F) (Figure 7C). Also by FITC-dextran assay, in BHI+FCS medium strains 208.41 (Figure 8A) and 106.66 (Figure 8B) expressed similar amounts of capsule. In MLM strain 106.66 (6B) (Figure 8D) bacteria are bigger than strain 208.41 (Figure 8C).

**Figure 9:**
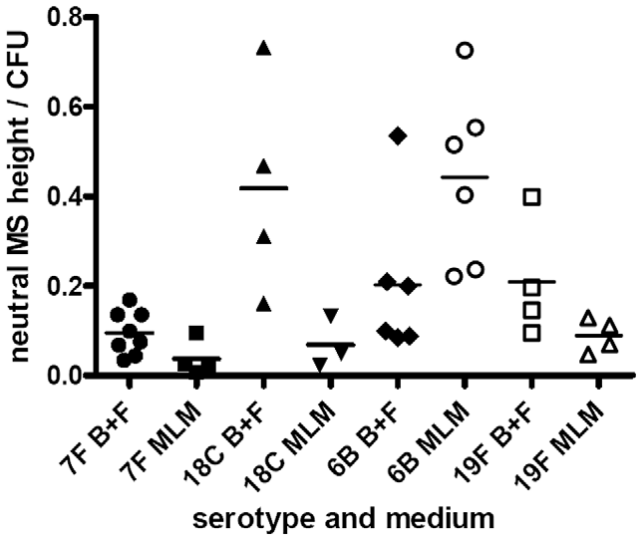
Quantification of capsule by HPLC. Cell bound capsular polysaccharides were released from wildtype strains and capsule switch mutants and after hydrolysis resulting in monosaccharides were quantified by reverse phase HPLC. The data was pooled by serotype.

For quantitative analysis, data was pooled for wildtypes strains and capsule switch mutants of the same serotype as shown in Figures 7E, 8E and 9. Comparisons were made between the low carriage prevalence serotypes 7F and 18C and the high prevalence serotypes 6B and 19F. Comparing capsule thickness in the two media, for the 7F/18C group it was clear that the amount of capsule was much reduced in the nutrient-restricted MLM compared with BHI+FCS (p<0.0001) when determined either by EM (Figure 7E) or FITC-dextran exclusion assay (Figure 8E) and the same trend was seen by HPLC (p = 0.0763) (Figure 9). For the 6B/19F group by FITC-dextran assay there was also more capsule in BHI+FCS than MLM (p<0.0001) (Figure 8E) but by EM (Figure 7E) and HPLC (Figure 9) the difference was not significant (p = 0.0664 and p = 0.2877 respectively) indicating that the nutrient-restricted medium has a less dramatic effect on reduction of capsule thickness in the high prevalence serotypes 6B and 19F. Comparing capsule thickness between the serotypes within the same medium, in the nutrient-restricted MLM there was significantly more capsule in the 6B/19F group whether measured by EM (Figure 7E) or FITC-dextran assay (Figure 8E) (p<0.0001) or by HPLC (Figure 9) (p = 0.0144). However, in BHI+FCS medium capsule thickness was greater in the 7F/18C group when measured by EM (Figure 7E) (p<0.0001) but greater in the 6B/19F group when measured by FITC-dextran assay (Figure 8E) (p<0.0001) and almost equivalent when measured by HPLC (Figure 9) (p = 0.9766) which may mean that in this rich medium capsule thickness is similar for the different serotypes.

### *cpsA* gene expression is lower in a nutritionally limiting environment especially in serotypes with high carriage prevalence

**Figure 10:**
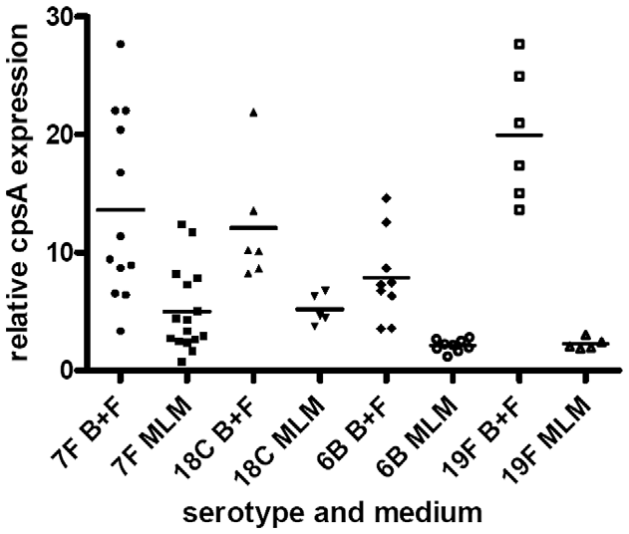
Relative *cpsA* expression. *CpsA* expression is displayed as the value for each isolate relative to that of the isolate with the lowest expression, after normalization using 16S RNA gene expression in BHI+FCS (B+F) medium and in MLM.

To address whether the differences observed in amount of capsule were regulated by transcription of the capsule operon, expression of the first gene, *cpsA*, was measured by real-time RT-PCR and normalized against 16S rRNA gene expression for the same strains as described in the capsule thickness experiments above. Figure 10 shows that after pooling data for wildtype and capsule switch mutants of the same serotype, for all serotypes tested *cpsA* expression was greater in the BHI+FCS medium than in the MLM (for 6B/19F p<0.0001, for 7F/18C p = 0.0375). In MLM *cpsA* expression was lower for the 6B/19F serotypes than the 7F/18C (p = 0.0016). There was no significant difference between the 7F and 18C versus the 6B and 19F in the BHI+FCS (p = 0.8669). The reduction in *cpsA* expression when growing in the MLM as compared to BHI+FCS was particularly marked for the high carriage prevalence 6B and 19F serotypes (p<0.0001) and to a lesser extent for the low carriage prevalence 7F and 18C serotypes (p = 0.0375). Therefore, expression levels of the *cpsA* gene agreed with capsule thickness in terms of being higher in the rich BHI+FCS medium as compared to the MLM. However, in MLM where differences between high and low carriage strains were most apparent, the high carriage prevalence serotypes 6B and 19F downregulated *cpsA* expression more than the low prevalence serotypes 7F and 18C although capsule thickness was higher in the high carriage prevalence strains than in the low carriage prevalence strains.

## Discussion

Although it has long been observed that some capsule serotypes of *S. pneumoniae* are most commonly isolated from the nasopharynx while others are predominantly found in normally sterile sites in patients with invasive disease [5]–[7], the reason for this difference has remained unclear. The role of the capsule is thought to be to protect the bacteria from phagocytosis following invasion [3], [4] and in the nasopharynx to repel mucus and so aid colonization [12]. It seems that the capsule type either enables a pneumococcus to reside for a long time in the nasopharynx (high colonization prevalence serotype) or causes it to be cleared quickly from the nasopharynx necessitating invasion for its survival (low colonization prevalence serotype). We speculate that this is because high colonization prevalence serotypes can thrive in the nasopharynx where as low colonization serotypes can only survive after invading and reaching a nutritionally richer environment. Weinberger et al. [7] have suggested that polysaccharide capsule serotypes differ in terms of the metabolic demand placed on the bacteria for their synthesis. They described an inverse correlation between the number of carbons and high energy bonds and colonization prevalence. Here we aimed to answer the question of whether serotypes predicted to have a low metabolic demand for their synthesis have high colonization prevalence because they have a growth advantage. Therefore, we compared growth phenotype of different serotypes and found that capsules predicted to be a low metabolic burden do have a growth advantage in terms of a short lag phase and a higher maximum optical density. These experiments were performed in a modified version of Lacks medium which we cannot be sure is an accurate representation of the nutrient conditions in the nasopharynx but rather we assume is closer to the nasopharyngeal conditions than rich media such as brain heart infusion broth or Todd Hewitt broth enriched with yeast extract often used to culture *S. pneumoniae*. Our version of the medium is limited in amino acids and peptides compared with the original Lacks medium in an attempt to mimic the nasopharyngeal environment which albumin concentration measurements predict to be more protein restricted than serum [11].

The growth advantage of having a capsule predicted to be of low metabolic burden correlated with ability to colonize the nasopharynx in a mouse model although a limitation of the in vivo study was that it was restricted to serotype 7F and 6B wildtype strains and their mutants. In addition, colonization was only monitored during the first week after colonization while colonization in the human nasopharynx occurs over a much longer period. A further limitation is that we have not determined whether differences in adherence to the epithelial cells play a role in the serotype-specific difference in colonization efficiency along with difference in growth.

Also, adding experimental evidence to the prediction made by Weinberger we saw that in the nutrient-restricted medium more energy was required to synthesize the capsules of the low colonization prevalence serotypes than the high colonization prevalence serotypes. In addition, we found that in the nutrient-restricted medium, serotypes with high colonization prevalence were able to make thicker capsules than the low colonization prevalence serotypes, as determined by electron microscopy, FITC-dextran exclusion assay and HPLC. This seems compatible with the idea that these capsules are less metabolically demanding for the bacteria and so more can be made. A thicker capsule could give these bacteria an advantage in resisting phagocytosis and clearance by mucus in the nasopharynx. Interestingly, the observation that high colonization serotypes make more capsule was not reflected in expression of the first gene of the capsule operon, *cpsA*, which in fact was slightly greater in the low colonization serotypes. This is in agreement with our previous study [10] which found an inverse correlation in BHI medium between *cpsA* expression and colonization prevalence. This raises the possibility that there is post-transcriptional control of capsule expression. A limitation of the current study is that we have not yet determined the mechanism of post-transcriptional control but the role of tyrosine phosphorylation of the product of *cpsD*, on capsular polysaccharide synthesis has already been described [13], [14]. *CpsC* and *cpsD* may regulate amount of capsule by affecting chain length of the polysaccharide polymers [13], [14]. The relationship between CpsD phosphorylation and amount of capsule produced appears to be affected by the genetic background of the bacteria suggesting that a factor outside the capsule operon is also important in the control of capsule synthesis [14]. In this study, although we describe a relationship between capsule type and capsule thickness we do not exclude that there is also control of capsule expression by factors outside of the capsule operon.

However, *cpsA* expression was affected by the nutrient availability in the medium: In the nutrient rich (BHI+FCS) medium there was more *cpsA* expression and more capsule production than in the nutrient-restricted (MEM) medium suggesting that regulation of capsule expression at the level of transcription also occurs.

In conclusion, in a nutrient-restricted environment, which might reflect the environment in the nasopharynx, serotypes that have a capsule which is not metabolically demanding to synthesize may be able to grow well and sooner and make a thicker capsule than serotypes with a metabolically demanding capsule. This regulation may be, to a large extent, by a post-transcriptional mechanism and give them an advantage in nasopharyngeal colonization. This may explain why some serotypes such as 6B, 19F, are 23F more often found colonizing the nasopharynx than serotypes such as 7F, 15 and 18C.

## Materials and Methods

### Ethics statement

All mouse experiments were conducted following guidelines from University of Liverpool ethical review and animal welfare committee. The mouse experiments were carried out under the authority of the UK Home Office Animals Scientific Procedures Act 1986. UK Home Office project Licence number PPL 80/2111, personal licence number PIL 70/13633, study approved by University of Liverpool Animal Welfare and Ethics Committee.

### Mouse strains

Outbred, female MF1 mice (Harlan, UK) at 8–12 weeks of age were used to model pneumococcal nasopharyngeal carriage [15], [16].

### Carriage model

Mice were mildly anaesthetized with 2.5% (v/v) Isofluorane USP (Isocare) over oxygen (1.4–1.6 litres/min) and 10 µl sterile PBS containing 1×10^5^ *S. pneumoniae* was equally distributed between both nostrils. At predetermined times, groups of 5 animals were sacrificed by cervical dislocation and nasopharyngeal tissue, was dissected [17], placed into 5 ml sterile PBS, weighed, disrupted with an Ultra-Turrax T8 homogeniser (IKA) and numbers of pneumococci determined by colony counting [18].

### Bacterial strains

Clinical isolates of *Streptococcus pneumoniae* were selected from two nationwide surveillance programs collecting nasopharyngeal and invasive isolates [6], [19]. Strains and mutants used for this study are listed in Tables S1 and S2.

### Construction of capsule switch mutants

For 10 wildtype clinical isolates the capsule operon was removed and replaced with a Janus cassette, as described below, rendering these strains non-typeable. These strains are listed in Table S1 and represent 7 different serotypes and 8 different genetic backgrounds. Capsule operons from other strains were introduced into these non-typeable mutants to give capsule switch mutants as described below. These mutants are listed in Table S2. In total, 81 different capsule switch mutants were used in this study representing 10 different serotypes.

For the construction of capsule switch mutants the bicistronic cassette Janus was used [20], [21]. Primers used for mutant construction are listed in Table S3. The Janus cassette in the form of a PCR construct of *dexB*-Janus-*aliA* was kindly provided by K. Trczinski, (Harvard School of Public Health, Boston, USA). The allele *rpsL str1*, which was kindly provided by D. Morrison (University of Illinois, Chicago, USA) was amplified with forward primer DAM350 and reverse Primer DAM351 by using Fast Taq DNA polymerase (Roche Molecular Biochemicals, Rotkreuz, Switzerland) according to the manufacturer's instructions. Amplification was performed by using the following cycling conditions: primary denaturation for 5 min at 94°C, followed by 30 cycles consisting of 94°C for 30 s, 50°C (annealing temperature) for 30 s and 72°C for 2 min (extension time) and then the last cycle for 10 min at 72°C.

The Janus cassette was amplified with forward primer dexBstart2 and reverse Primer aliAend2 by using the Expand Long Template PCR system (LRPCR) (Roche Molecular Biochemicals, Rotkreuz, Switzerland). Amplification was performed by using the following cycling conditions: primary denaturation for 2 min at 92°C, followed by 10 cycles consisting of 92°C for 10 s, 65°C for 30 s, and 68°C for 17 min and then 20 cycles in which each extension cycle was prolonged by 20 s. PCR products were purified with a QIAquick PCR purification kit (QIAGEN, Basel, Switzerland). The *dexB-Janus-aliA* PCR product was used to transform pneumococcal clinical isolates with selection for Km^r^ to make the unencapsulated strains, which were used as recipients. Chromosomal DNA of the “capsule donor” clinical isolates was used to transform the recipients with selection for Sm^r^ to create capsule switch mutants. The structure of the *cps* locus and surrounding regions of the capsule switch mutants were confirmed by RLFP analysis of the following PCR products: 1430-1402 (*dexB-cps-aliA* locus) digested with *Rsa*I, TTM07-09 (*pbp2x-dexB* flanking *cps* upstream region) and TTM08-10 (*aliA-pbp1a* flanking *cps* downstream region) both digested with *Tsp*509I [21]. Restriction patterns were compared with recipient and donor strain.

Transformation was performed as described previously [22] using 1 µg of DNA consisting of the *rpsL str1* DNA fragment, *dexB-Janus-aliA* PCR product, or 2 µg of the chromosomal DNA of the donor strain. Aliquots of the cultures were then spread onto CSBA plates containing 300 µg/ml streptomycin or 500 µg/ml kanamycin. The plates were incubated for 24 h prior to subculture of single colonies on CSBA plates. After serotyping, successful transformants were stored for further evaluation at −80°C using Protect bacterial preservers (Technical Service consultants, Heywood, UK).

### Growth media and measurement of growth

Two media were used to study the growth behaviour of the bacteria. To represent a rich nutritional environment, brain heart infusion broth (BHI) (Becton Dickinson and Company, le Pont de Claix, France) supplemented with 5% foetal calf serum (FCS) (Biochrom KG, Berlin, Germany) was used. This is referred to in the text as BHI+FCS medium. To represent a more nutritionally limited environment a modified version of Lacks medium was used [23]. The principal modification from the original medium is that the amino acid and peptide components are at one quarter of the concentration of the original medium. The components of modified Lacks medium (MLM) are listed in Table S4.

Strains were streaked onto Columbia sheep blood agar (CSBA) plates and incubated at 37°C in a 5% CO_2_-enriched atmosphere overnight then subcultured in growth medium to OD_600_ 0.5, centrifuged at 3000 g for 5 min and resuspended in growth medium. Growth was monitored in sterile flat-bottomed 96-well microtitre plates (Nunclon Surface, Nunc, Denmark) based on the method of Brewster [24]. In brief, 200 µl bacteria culture was grown per well and OD_450 nm_ measured every 30 minutes using a VERSAmax microplate reader (Molecular Devices) over 20 hours. The plate was shaken automatically for 5 seconds before each reading. The problem of condensation affecting the readings was avoided by pre-treating the lids of the 96-well plates with 3 ml 0.05% Triton X-100 in 20% ethanol and allowing them to air-dry before use.

### Colony forming unit (CFU) quantification

Serial dilutions of 100 µl liquid culture in phosphate buffered saline (PBS) were plated out onto CSBA plates at least in duplicates and incubated overnight at 37°C with 5% CO_2_ atmosphere. The next day CFUs were counted with an in house automated colony counter (Brugger S. et al., unpublished data) and the bacterial load was calculated.

### Energy deposition into the capsule

Strains were grown overnight on Columbia blood sheep agar (CSBA) plates at 37°C with 5% CO_2_ atmosphere. Several colonies were inoculated into 10 ml of growth medium (BHI+FCS or MLM). The next day, 100 µl of the overnight culture in BHI+FCS was inoculated into 10 ml of fresh BHI+FCS medium and 100 µl of the overnight culture in MLM was inoculated into 10 ml of fresh MLM, both supplemented with 1 µCi D-glucose-3H(U) (ARC, St. Louis, MO). The subcultures were grown to an optical density (OD) at 600 nm wavelength between 0.4–0.5 representing mid-log phase of growth. Bacteria were harvested by centrifugation at 4000× G for 10 minutes at 4°C and washed with 5 ml phosphate buffered saline (PBS). After ultracentrifugation with 20000× G for 30 minutes at 4°C the bacterial pellet was dissolved in 5 ml double distilled water, buffer saturated phenol was added to a final concentration of 1% and incubated overnight at room temperature. The released capsular polysaccharides were separated from the cells and detritus by centrifugation with 20000× G for 30 minutes at 4°C. The supernatant containing the capsular polysaccharides was decanted and the cells resuspended in 5 ml of double distilled water. Radioactivity was determined in the collected cell and capsule fraction (see above). Therefore, 500 µl of each fraction was mixed with 1500 µl Microscint 40 scintillation liquid and counts per minute were measured averaging 3 minutes per sample with a Tri-Carb liquid scintillation counter (Perkin Elmer, Waltham, MA). A capsule/cell beta counts ratio was calculated for each tested strain to compare the energy distribution for capsule assembly and cellular metabolism.

### Quantification of capsule production

High performance liquid chromatography: Isolation of polysaccharide capsule was done using a procedure adapted from previously described methods [25], [26]. Bacteria were harvested by centrifugation and washed twice with phosphate buffer saline (PBS) and double distilled water. After ultracentrifugation at 20000 g for 30 minutes at 4°C the pellet was dissolved in a 1% buffer-saturated phenol solution. This mixture was incubated overnight at room temperature to release cell bound capsular polysaccharides. The capsular polysaccharides were separated from the cells by ultracentrifugation at 20000 g for 30 minutes at 4°C. Polysaccharides were precipitated by adding sodium acetate and ethanol to a final concentration of 7.2% and 60%, respectively and incubation for at least 30 minutes at 4°C. The polysaccharides were collected by centrifugation at 20000 g for 30 minutes and remaining nucleic acids and proteins were digested by the sequential addition of 250 U of Benzonase nuclease (Merck, Darmstadt, Germany) (4 hours incubation) and 40 µl of a 20 mg/ml proteinase K solution (Roche, Mannheim, Germany) (incubated overnight). Resulting low-molecular weight contaminants were removed by centrifugation in an Amicon Ultra 30 kDa cut off membrane centrifugal filter unit (Millipore, Billerica, MA) at 4000 g for 10 minutes at 4°C. The retained polysaccharides were dried with heat in speed vac vacuum centrifuge. After resolution, extracted capsular polysaccharides were hydrolysed as described previously (ZITAT Saddic/ANumula). After complete hydrolysis, the dried hydrolysed polysaccharides (i.e. monosaccharides) were labelled with anthranilic acid (Fluka, Buchs, Switzerland) to enable fluorescence detection in HPLC separation. Labelling was performed as described previously [27]–[29].

Chromatographic separation was adapted from a previously described method [29] using an AS2000A autosampler (Merck Hitachi, Darmstadt, Germany) with an injection volume of 20 µl per sample. Separation of the monosaccharide was done with a flow rate of 0.85 ml/min as follows: 6% solvent B isocratic for 35 minutes followed by a linear gradient from 6 to 12% solvent B over 20 minutes. Subsequently, a ten minute wash with 100% B and 100% A for 15 minutes followed before re-equilibrating the system with 6% B for minutes. Total run time was 85 minutes with data collection for 60 minutes. A Luna 5 µm, C18, 150×4.6 mm column (Phenomenex, Torrance, CA) was used for separation and column temperature was maintained at 35°C using a L5025 column oven (Merck Hitachi).

Anthranilic acid labelled monosaccharides were detected by fluorescence (F-1080 Fluorescence Detector, Merck Hitachi) at an excitation of 360 nm and an emission of 425 nm. Peaks were identified by comparison of retention times with monosaccharide standards and re-running after spiking to verify increase in the peak of interest. Standard curves were generated for monosaccharides of interest in at least three independent experiments. Amounts of monosaccharides per bacterial cells were calculated using peak heights and/or areas taking into account dilution factors, sample and injection volumes used and relating to counted CFUs (see above).

#### Electronmicroscopy

Strains were grown as described above and harvested by centrifugation after reaching mid-log phase OD. Cryofixation was achieved by high-pressure freezing as described before [30]. To prevent freezing artifacts, pellets were dissolved 1∶1 in the corresponding growth medium supplemented with 20% dextrane and 5% sucrose. Ruthenium red was used to enhance capsule resolution [31]. Capsule size was calculated from EM pictures as described by Hyams et al. [32] after blinding.

#### FITC-dextran exclusion

Capsule thickness was also determined by measuring the zone of exclusion of FITC-dextran based on the method of Gates et al. [33] using FITC-dextran of 2000 kDa (Sigma). Bacteria were cultured overnight in BHI+FCS medium or MLM until OD_600 nm_ = 0.3 then 200 µl subcultured into 10 ml of fresh medium and cultured again until OD_600 nm_ 0.3. After centrifuging at 3000 g for 5 minutes the pellet was resuspended in 500 µl of PBS. 10 µl bacterial suspension was mixed with 2 µl FITC-dextran (10 mg/ml in water), pipetted onto a microscope slide and a coverslip applied firmly. Each strain was prepared twice on different days. The slides were viewed using a Zeiss Axio Imager.M1 fluorescence microscope with a 100× objective and photographed by a Zeiss AxioCam HRc camera. The images were converted to greyscale and analyzed with UTHSCSA ImageTool for Windows v3.0 (University of Texas Health Science Center in San Antonio) software. A brightfield image was also photographed for each fluorescent image recorded in order to count the number of bacteria per chain. For each strain the mean area of between 47 and 247 bacteria was determined after blinding. SAS 9.2 for Windows (SAS Institute Inc., Cary, NC, USA) was used for the statistical analysis.

### Expression of the capsule operon

Bacteria were cultured overnight in BHI+FCS medium or MLM until OD_600 nm_ 0.3 then 200 µl subcultured into 10 ml of fresh medium and cultured again until OD_600 nm_ 0.3. After addition of 20 ml RNAprotect (Qiagen), RNA was extracted and expression of the first gene of the capsule operon, *cpsA*, was determined by real-time RT-PCR as described previously [10].

### Statistical analysis

Student's t-test was used to calculate p values using the software GraphPad Prism (Version 5, GraphPad Software, Inc.). This software was also used to calculate linear regression. A value of p≤0.05, two-tailed, was considered significant.

## Supporting Information

Figure S1 **A second example of growth patterns of strains of serotypes 7F and 6B and their capsule switch mutants.** A) Loss of 7F capsule greatly increases growth and acquisition of a 6B capsule causes only slightly increase the lag phase compared to the NT Janus mutant and enhances maximum OD_450 nm_ in MLM. B) Loss of 6B capsule reduces maximum OD_450 nm_ and slightly increases lag phase in MLM. Acquisition of a 7F capsule further enhances these effects. C) In BHI+FCS medium there is little difference in lag phase between the strains or maximum OD_450 nm_ between the wildtype strain. Graphs are representative of three independent experiments.
(TIF)

Figure S2 **Relationship between delay of growth in MLM due to capsule and carriage prevalence of serotype.** For each serotype, the delay of growth due to the capsule was plotted against percentage carriage prevalence data obtained from A) a local study [6], B) Weinberger et al. [7]. In both cases an inverse correlation was found which was statistically significant with p = 0.0387 and p = 0.0379 respectively.
(TIF)

Figure S3 **Relationship between difference in maximum OD_450 nm_ in MLM due to capsule and carriage prevalence of serotype.** For each serotype, the difference in maximum OD_450 nm_ due to the capsule was plotted against percentage carriage prevalence data obtained from A) a local study [6], B) Weinberger et al. [7]. In both cases a positive correlation was found which for A and was statistically significant (p = 0.0361) and for C gave a p value of 0.0511.
(TIF)

Table S1 **Wildtype clinical isolates from which non-typeable Janus mutants were constructed.**
(PDF)

Table S2 **Capsule switch mutants.**
(PDF)

Table S3 **Primers used in construction of capsule switch mutants.**
(PDF)

Table S4 **Composition of modified Lacks medium (MLM).**
(PDF)
