# Supplementary figures and images for: Capsule Type of Streptococcus pneumoniae Determines Growth Phenotype
Source: PLoS Pathog. 2012 Mar 8;8(3):e1002574. doi: 10.1371/journal.ppat.1002574 (PMC3297593; doi:10.1371/journal.ppat.1002574)

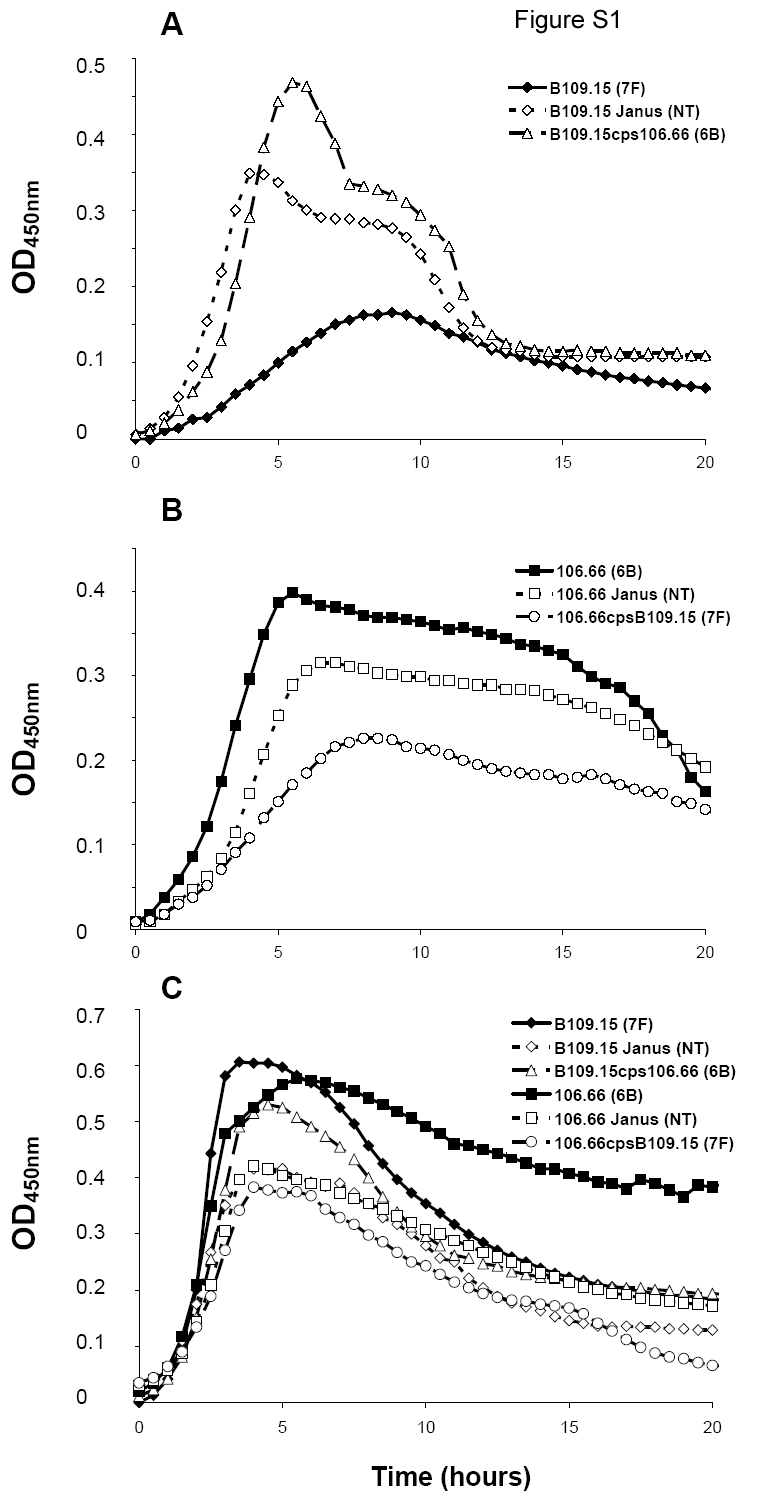

Supplement: Figure S1 — A second example of growth patterns of strains of serotypes 7F and 6B and their capsule switch mutants. A) Loss of 7F capsule greatly increases growth and acquisition of a 6B capsule causes only slightly increase the lag phase compared to the NT Janus mutant and enhances maximum OD450 nm in MLM. B) Loss of 6B capsule reduces maximum OD450 nm and slightly increases lag phase in MLM. Acquisition of a 7F capsule further enhances these effects. C) In BHI+FCS medium there is little difference in lag phase between the strains or maximum OD450 nm between the wildtype strain. Graphs are representative of three independent experiments. (TIF) [file ppat.1002574.s001.tif]

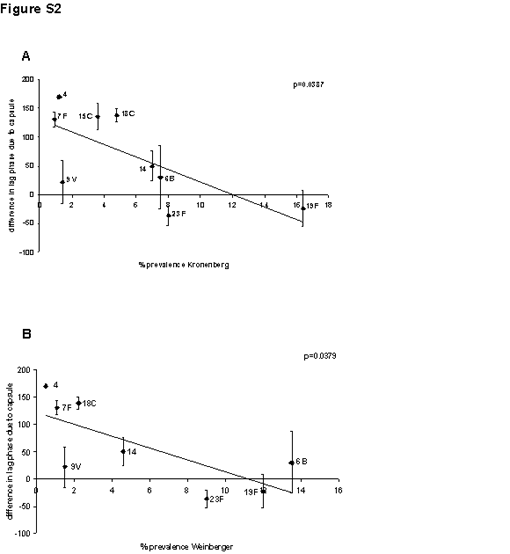

Supplement: Figure S2 — Relationship between delay of growth in MLM due to capsule and carriage prevalence of serotype. For each serotype, the delay of growth due to the capsule was plotted against percentage carriage prevalence data obtained from A) a local study [6], B) Weinberger et al. [7]. In both cases an inverse correlation was found which was statistically significant with p = 0.0387 and p = 0.0379 respectively. (TIF) [file ppat.1002574.s002.tif]

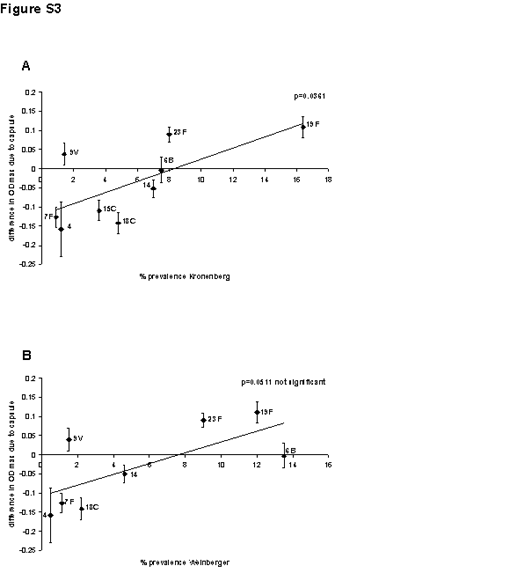

Supplement: Figure S3 — Relationship between difference in maximum OD450 nm in MLM due to capsule and carriage prevalence of serotype. For each serotype, the difference in maximum OD450 nm due to the capsule was plotted against percentage carriage prevalence data obtained from A) a local study [6], B) Weinberger et al. [7]. In both cases a positive correlation was found which for A and was statistically significant (p = 0.0361) and for C gave a p value of 0.0511. (TIF) [file ppat.1002574.s003.tif]
