# Supplementary material for: Capsule Type of Streptococcus pneumoniae Determines Growth Phenotype
Source: PLoS Pathog. 2012 Mar 8;8(3):e1002574. doi: 10.1371/journal.ppat.1002574 (PMC3297593; doi:10.1371/journal.ppat.1002574)
Supplement: Table S1 — Wildtype clinical isolates from which non-typeable Janus mutants were constructed. (PDF) [file ppat.1002574.s004.pdf]

**Table S1 - Wildtype clinical isolates from which non-typeable Janus mutants were constructed**

| Strain | Origin | Diagnosis | Serotype | RFLP* | Capsule size (bp) | MLST |
|---|---|---|---|---|---|---|
| 103.57 | nasopharynx | otitis media | 23F | 11 | 22330 | 507 |
| 106.66 | nasopharynx | otitis media | 6B | 3 | 17506 | 2244 |
| 208.41 | nasopharynx | otitis media | 7F | 8 | 24127 | 191 |
| 307.14 | nasopharynx | otitis media | 18C | 14 | 21819 | 113 |
| B101.38 | blood | pneumonia | 5 | 28 | 19969 | 289 |
| B103.66 | blood | | 14 | 1 | 19918 | ¶ |
| B109.15 | blood | pneumonia | 7F | 8 | 24127 | 191 |
| B110.04 | blood | | 7F | 8 | 24127 | 191 |
| B201.61 | blood | | 18C | 16 | 21819 | 1233 |
| B201.73 | blood | | 19F | 40 | 19798 | 43 |

*RFLP data was obtained as described in Hathaway LJ, Brugger S, Martynova A, Aebi S, Mühlemann K (2007) Use of the Agilent 2100 bioanalyzer for rapid and reproducible molecular typing of Streptococcus pneumoniae. J Clin Microbiol. 45: 803-9.

¶ Novel MLST: *aroE* allele 7, *gdh* allele 121, *gki* allele 10, *recP* allele 4, *spi* allele 6, *xpt* allele 58, *ddl* new allele with 99% match to allele 286.
