# Supplementary material for: Capsule Type of Streptococcus pneumoniae Determines Growth Phenotype
Source: PLoS Pathog. 2012 Mar 8;8(3):e1002574. doi: 10.1371/journal.ppat.1002574 (PMC3297593; doi:10.1371/journal.ppat.1002574)
Supplement: Table S2 — Capsule switch mutants. (PDF) [file ppat.1002574.s005.pdf]

Table S2 - Capsule switch mutants

Recipient				Donor				Capsule switch mutant	
Strain	Sero-type	RFLP	Capsule size (bp)	Strain	Sero-type	RFLP	Capsule size (bp)	Mutant	Sero-type
103.57	23F	11	22330	103.57	23F	11	22330	103.57cps103.57	23F
103.57	23F	11	22330	203.29	23F	11	22330	103.57cps203.29	23F
106.66	6B	3	17506	106.66	6B	3	17506	106.66cps106.66	6B
106.66	6B	3	17506	208.41	7F	8	24127	106.66cps208.41	7F
106.66	6B	3	17506	B109.15	7F	8	24127	106.66cpsB109.15	7F
106.66	6B	3	17506	B101.77	14	8	19918	106.66cpsB101.77	14
106.66	6B	3	17506	201.38	9V	1	20856	106.66cps201.38	9V
106.66	6B	3	17506	109.74	9V	1	20856	106.66cps109.74	9V
106.66	6B	3	17506	207.31	15	1	18626	106.66cps207.31	15
106.66	6B	3	17506	307.14	18C	14	21819	106.66cps307.14	18C
106.66	6B	3	17506	B112.27	18C	14	21819	106.66cpsB112.27	18C
106.66	6B	3	17506	103.57	23F	11	22330	106.66cps103.57	23F
106.66	6B	3	17506	108.34	19F	39	19798	106.66cps108.34	19F
106.66	6B	3	17506	111.46	19F	4	19798	106.66cps111.46	19F
106.66	6B	3	17506	201.47	19F	7	19798	106.66cps201.47	19F
106.66	6B	3	17506	B201.73	19F	40	19798	106.66cpsB201.73	19F
208.41	7F	8	24127	208.41	7F	8	24127	208.41cps208.41	7F
208.41	7F	8	24127	B109.15	7F	8	24127	208.41cpsB109.15	7F
208.41	7F	8	24127	B204.27	4	17	20936	208.41cpsB204.27	4
208.41	7F	8	24127	201.12	14	5	19918	208.41cps201.12	14
208.41	7F	8	24127	B103.66	14	1	19918	208.41cpsB103.66	14
208.41	7F	8	24127	109.74	9V	1	20856	208.41cps109.74	9V
208.41	7F	8	24127	201.38	9V	1	20856	208.41cps201.38	9V
208.41	7F	8	24127	106.66	6B	3	17506	208.41cps106.66	6B
208.41	7F	8	24127	108.34	19F	39	19798	208.41cps108.34	19F
208.41	7F	8	24127	B201.73	19F	40	19798	208.41cpsB201.73	19F

Table S2 (continued)

208.41	7F	8	24127	103.57	23F	11	22330	208.41cps103.57	23F
208.41	7F	8	24127	203.29	23F	11	22330	208.41cps203.29	23F
208.41	7F	8	24127	B201.61	18C	16	21819	208.41cpsB201.61	18C
208.41	7F	8	24127	307.14	18C	14	21819	208.41cps307.14	18C
307.14	18C	14	21819	208.41	7F	8	24127	307.14cps208.41	7F
307.14	18C	14	21819	B101.77	14	8	19918	307.14cpsB101.77	14
B101.38	5	28	19969	208.41	7F	8	24127	B101.38cps208.41	7F
B101.38	5	28	19969	203.39	4	17	20936	B101.38cps203.39	4
B101.38	5	28	19969	201.12	14	5	19918	B101.38cps201.12	14
B101.38	5	28	19969	B101.77	14	8	19918	B101.38cpsB101.77	14
B101.38	5	28	19969	109.74	9V	1	20856	B101.38cps109.74	9V
B101.38	5	28	19969	201.38	9V	1	20856	B101.38cps201.38	9V
B101.38	5	28	19969	106.66	6B	3	17506	B101.38cps106.66	6B
B101.38	5	28	19969	108.34	19F	39	19798	B101.38cps108.34	19F
B101.38	5	28	19969	B201.73	19F	40	19798	B101.38cpsB201.73	19F
B101.38	5	28	19969	203.29	23F	11	22330	B101.38cps203.29	23F
B101.38	5	28	19969	103.57	23F	11	22330	B101.38cps103.57	23F
B101.38	5	28	19969	B201.61	18C	16	21819	B101.38cpsB201.61	18C
B101.38	5	28	19969	307.14	18C	14	21819	B101.38cps307.14	18C
B101.38	5	28	19969	207.31	15	1	18626	B101.38cps207.31	15
B103.66	14	1	19918	B103.66	14	1	19918	B103.66cpsB103.66	14
B103.66	14	1	19918	201.12	14	5	19918	B103.66cps201.12	14
B103.66	14	1	19918	106.66	6B	3	17506	B103.66cps106.66	6B
B103.66	14	1	19918	B201.73	19F	40	19798	B103.66cpsB201.73	19F
B103.66	14	1	19918	B112.27	18C	14	21819	B103.66cpsB112.27	18C
B103.66	14	1	19918	207.31	15	1	18626	B103.66cps207.31	15
B109.15	7F	8	24127	B109.15	7F	8	24127	B109.15cpsB109.15	7F
B109.15	7F	8	24127	106.66	6B	3	17506	B109.15cps106.66	6B
B109.15	7F	8	24127	108.34	19F	39	19798	B109.15cps108.34	19F

Table S2 (continued)

B109.15	7F	8	24127	103.57	23F	11	22330	B109.15cps103.57	23F
B109.15	7F	8	24127	203.29	23F	11	22330	B109.15cps203.29	23F
B109.15	7F	8	24127	B201.61	18C	16	21819	B109.15cpsB201.61	18C
B109.15	7F	8	24127	307.14	18C	14	21819	B109.15cps307.14	18C
B109.15	7F	8	24127	B201.73	19F	40	19798	B109.15cpsB201.73	19F
B109.15	7F	8	24127	207.31	15	1	18626	B109.15cps207.31	15
B110.04	7F	8	24127	B201.73	19F	40	19798	B110.04cpsB201.73	19F
B110.04	7F	8	24127	203.29	23F	11	22330	B110.04cps203.29	23F
B110.04	7F	8	24127	B112.27	18C	14	21819	B110.04cpsB112.27	18C
B201.61	18C	16	21819	B101.77	14	8	19918	B201.61cpsB101.77	14
B201.61	18C	16	21819	109.74	9V	1	20856	B201.61cps109.74	9V
B201.61	18C	16	21819	203.29	23F	11	22330	B201.61cps203.29	23F
B201.73	19F	40	19798	208.41	7F	8	24127	B201.73cps208.41	7F
B201.73	19F	40	19798	B101.77	14	8	19918	B201.73cpsB101.77	14
B201.73	19F	40	19798	201.12	14	5	19918	B201.73cps201.12	14
B201.73	19F	40	19798	201.38	9V	1	20856	B201.73cps201.38	9V
B201.73	19F	40	19798	109.74	9V	1	20856	B201.73cps109.74	9V
B201.73	19F	40	19798	203.39	4	17	20936	B201.73cps203.39	4
B201.73	19F	40	19798	106.66	6B	3	17506	B201.73cps106.66	6B
B201.73	19F	40	19798	103.57	23F	11	22330	B201.73cps103.57	23F
B201.73	19F	40	19798	203.29	23F	11	22330	B201.73cps203.29	23F
B201.73	19F	40	19798	B112.27	18C	14	21819	B201.73cpsB112.27	18C
B201.73	19F	40	19798	B201.61	18C	16	21819	B201.73cpsB201.61	18C
B201.73	19F	40	19798	307.14	18C	14	21819	B201.73cps307.14	18C
B201.73	19F	40	19798	207.31	15	1	18626	B201.73cps207.31	15
B201.73	19F	40	19798	B201.73	19F	40	19798	B201.73cpsB201.73	19F
