# Supplementary material for: Capsule Type of Streptococcus pneumoniae Determines Growth Phenotype
Source: PLoS Pathog. 2012 Mar 8;8(3):e1002574. doi: 10.1371/journal.ppat.1002574 (PMC3297593; doi:10.1371/journal.ppat.1002574)
Supplement: Table S3 — Primers used in construction of capsule switch mutants. (PDF) [file ppat.1002574.s006.pdf]

Table S3 – Primers used in construction of capsule switch mutants

Primer	Sequence	Description
dexB vorstart	ggatagtaatgaagatggagttggtga	Control of correct place of Janus cassette, LRPCR for control of correct ligation of capsule to upstream flanking region
kan_B1	gatattctcattttagccatttattatttc	Control of correct place of Janus cassette
kan_start_F1	aggaaataataaatggctaaaatgagaat	Control of correct size of Janus cassette
DAM351	ctttccttatgcttttggac	Control of correct size of Janus cassette. Sequencing primer for *str* allele
rpsL_mitte_B1	gaatttacgaagggctgagttaggttt	Control of intact Janus cassette and correct place of the Janus cassette
rpsL_mitte_F1	aaaaaacctaactcagcccttcgtaa	Control of correct place of the Janus cassette
DAM350	accaaaaataaaaaaacacaggag	Sequencing primer for *str* allele
dexBstart2	tttctcccgtttatgacagccctatgg	Amplification of Janus cassette
aliAend2	aagattggacgccctgtacgagatgt	Amplification of Janus cassette
aliAend1b	ctggttcacttgtacctttatttcct	Control of correct place of Janus cassette and control of correct ligation of capsule to downstream capsule flanking reagion
dexB-cpsA_REV	ctatctgctaaaacagcgacactga	LRPCR for control of correct ligation of capsule to upstream capsule flanking region
cpsO_F1	caaatggccaattaggaacgg	Control of correct ligation of capsule to downstream capsule flanking region
1430TRZ	tgtccaatgaagagcaagacttgacagtag	LRPCR detection of capsule operon and RFLP
1402TRZ	caattgtcacgcccgcaagggcaagt	LRPCR detection of capsule operon and RFLP
LRPdexB_vorstart1	tggatagtaatgaagatggagttggtgatttgco	LRPCR detection of Janus cassette
LRPaliA_end1	catgttttgcgagatcttcttgagccotttttatt	LRPCR detection of Janus cassette
TTM07_TRZ	ctactgttcaagtcttgctcttcattggaca	LRPCR detection of capsule upstream flanking region for RFLP

Table S3 (continued)

TTM09_TRZ	ctaaaacaggggaaattctggcaacaacgc	LRPCR detection of capsule upstream flanking region for RFLP
TTM08_TRZ	acttgcccttgcgggcgtgacattattg	LRPCR detection of capsule downstream flanking region for RFLP
TTM10_TRZ	aatcgcgaaacgtcccagccgtggaaactc	LRPCR detection of capsule downstream flanking region for RFLP
