# Supplementary material for: Capsule Type of Streptococcus pneumoniae Determines Growth Phenotype
Source: PLoS Pathog. 2012 Mar 8;8(3):e1002574. doi: 10.1371/journal.ppat.1002574 (PMC3297593; doi:10.1371/journal.ppat.1002574)
Supplement: Table S4 — Composition of modified Lacks medium (MLM). (PDF) [file ppat.1002574.s007.pdf]

Table S4 – Composition of modified Lacks medium (MLM) – a modification of the medium described by Lacks [1]

Component	Amount / litre
casamino acids (Bacto)	1.25 g
casein hydrolysate (Bacto)	0.25 g
tryptophan (Sigma)	1.5 mg
cysteine HCl (Merck)	10 mg
asparagine (Sigma)	12.5 mg
glutamine (Merck)	2.5 mg
choline HCl	5 mg
adenine	5 mg
sodium acetate	2 g
K_2HPO_4	8.5 g
$MgCl_2 \cdot 6\ H_2O$	0.5 g
$CaCl_2$	0.6 mg
$MnSO_4 \cdot 4\ H_2O$	0.2 mg
$FeSO_4 \cdot 7\ H_2O$	0.5 mg
$CuSO_4 \cdot 5\ H_2O$	0.5 mg
$ZnSO_4 \cdot 7\ H_2O$	0.5 mg
$NaHCO_3$	0.4 g
biotin	0.6 µg
nicotinic acid	0.3 mg
pyridoxine·HCl	0.3 mg
thiamine·HCl	0.3 mg
riboflavine	0.14 mg
calcium pantothenate	1.2 mg
sucrose	3.423 g
catalase from bovine liver (Sigma)	12000 units

pH adjusted to 7.3

1. Lacks S (1966) Integration efficiency and genetic recombination in pneumococcal transformation. Genetics 53: 207-235.
